# Implementation of lockdown, quarantine, and isolation measures in the context of COVID-19 among internally displaced persons in Burkina Faso: a qualitative study

**DOI:** 10.1186/s13031-024-00579-4

**Published:** 2024-03-01

**Authors:** Kadidiatou Kadio, Antarou Ly, Adidjata Ouédraogo, Mohamed Ali Ag Ahmed, Sanni Yaya, Marie-Pierre Gagnon

**Affiliations:** 1 Centre national de la recherche scientifique et technologique (CNRST)/Institut de recherche en sciences de la santé (IRSS), 03 BP 7047 Ouagadougou, Burkina Faso; 2 Département de médecine sociale et préventive, Université Laval, Québec, QC G1V 0A6 Canada; 3 Centre de recherche du CHU de Québec-Université Laval, Québec, QC G1S 4L8 Canada; 4 Institut de médecine tropicale d’Anvers, Kronenburgstraat 43, Antwerpen, 2000 Belgique; 5 Faculty of Social Sciences, University of Ottawa, 120 University Private, Ottawa, ON K1N 9A7 Canada; 6 Faculté des sciences infirmières, Université Laval, Québec, G1V 0A6 Canada; 7 Fellow Pilote African Postdoctrorat Academy – PAPA, Université Goethe de Francfort, Francfort, Hesse Allemagne

**Keywords:** Internally displaced persons, Covid-19, Lockdown, Burkina Faso, Sahel countries

## Abstract

**Background:**

The triple political, security, and health crisis in Burkina Faso has impacted the lives of Burkinabè people, resulting in massive internal displacement. These internally displaced persons (IDPs) are very vulnerable to epidemic diseases, which was exacerbated by the recent COVID-19 pandemic., The implementation of public health measures to curb the spread of COVID-19 represented a major concern among IDPs. The objective of this study was to document knowledge, difficulties, adjustments, and challenges faced by IDPs and humanitarian authorities/actors during implementation of lockdown, quarantine, and isolation measures in response to COVID-19.

**Methods:**

The study was conducted in Burkina Faso, in the north-central region Kaya, a commune which hosts the largest number of IDPs in the country. Qualitative research using semi-structured interviews collected discursive data from 18 authorities and/or humanitarian actors and 29 IDPs in June 2021. The transcribed interviews were coded with N’vivo 11 software and analyzed thematically.

**Results:**

Although respondents had a good knowledge of lockdown, isolation, and quarantine measures, the difference between these three concepts was not easily understood by either authorities/humanitarian actors or IDPs. Communication was one of the biggest challenges for humanitarian actors. The difficulties encountered by IDPs were economic (lack of financial resources), infrastructural (limited housing), and socio-cultural in the application of lockdown, isolation, and quarantine measures. As for adjustment measures, the health authorities developed a strategy for isolation and quarantine for the management of positive and suspected cases. The IDPs mentioned their commitment to compliance and awareness of lockdown measures as the main adjustment.

**Conclusion:**

Although there were no known cases of COVID-19 among the IDPs at the time of the study, tailored response plans were developed to facilitate the application of these measures in emergencies. The involvement of IDPs in the communication and sensitization process was necessary to facilitate their adherence to these different measures.

## Background

On March 9, 2020, Burkina Faso announced its first case of COVID-19 [1]. At the same time, the World Health Organization (WHO) declared COVID-19 a public health emergency of international concern and a threat of the highest rank [2]. This pandemic came at a time when Burkina Faso was experiencing one of the worst security crises in its history, marked by terrorist attacks [3, 4]. Indeed, it is the second most affected country by terrorist attacks in Africa [5]. This dual health and security crisis was further compounded by a political crisis marked by two military coups in 2022.

These multidimensional crises in Burkina Faso led to massive internal displacement, with the number of internally displaced persons (IDPs) that was 1.6 million in December 2021, [6].

Unlike refugees, the state is legally responsible for the protection and welfare of IDPs. Forced displacement is one of the most tragic incidents that can happen to individuals [7]. Compared to their host community, IDPs are more vulnerable on several socio-economic and health aspects because of their lack of decent housing (usually spontaneous, unsanitary, and cramped), insufficient support, and lack of information to promote healthy living [8]. Internal displacement is one of the most damaging human mobility problems and urban phenomena, both for the people affected and for the host city [8]. Yet, it remains largely unaddressed in international discourse, advocacy, and research [9].

IDPs are often forced to settle in densely populated areas. They often have limited or no access to basic services (health facilities, health promotion, sanitation, clean water), excluding them from most forms of aid and assistance [10]. This poor accessibility disrupts their socioeconomic stability and makes them vulnerable to health problems [11]. In most cases, health systems are unable to manage them in the face of severe and critical forms of diseases such as during a pandemic [12].

In Burkina Faso, poor people were disproportionately affected by restrictive measures to combat COVID-19 such as containment [13]. While COVID-19 requires several public health measures (hand washing, nose covering, physical distancing etc.), their application in such contexts is difficult, if not impossible, for some barrier measures like physical distancing measures. In an attempt to protect IDPs, who are at higher risk of COVID-19, the government of Burkina Faso, with the support of humanitarian actors, implemented actions to curb transmission and limit virus spread [14]. Among these response measures were isolation, quarantine, and lockdown, which are known to be effective in reducing transmission of the virus [12]. Isolation is the separation of individuals suspected or infected with COVID-19 from uninfected individuals [15]. Quarantine involves restricting the movement and close contact with suspected or infected patients, ideally combined with medical observation during the 14 day incubation period [16]. Lockdown is an intervention applied to an entire community, city, or region, designed to reduce personal interaction, except for minimal and protected interaction for essential needs [15]. Openshaw and Travassos [17] showed that the best preventive option against human-to-human transmission of COVID-19 among IDPs is adherence to public health policies, particularly isolation and quarantine measures. However, implementation of these measures to slow the spread of the virus was difficult among IDPs [18] due to inadequate housing conditions [8]. Recent studies in Burkina Faso showed that the living conditions and financial precariousness of IDPs and the lack of material and infrastructural resources for care increase the vulnerability of IDPs to HIV infection [18, 19]. Additionally, a study of IDPs in Mali found that lack of isolation and quarantine space, fear of stigma, and proximity were barriers to adopting isolation and quarantine measures [12]. This study aims to document the knowledge, difficulties, adjustments, and challenges faced by IDPs and humanitarian authorities/actors during implementation of lockdown, quarantine, and isolation measures in response to COVID-19 in North Central Region of Burkina Faso.

The purpose of this study is to: (1) explore the knowledge and challenges of implementing lockdown, isolation, and quarantine measures among IDPs and humanitarian authorities/actors; (2) describe the adjustments made by IDPs and humanitarian authorities/actors to overcome these difficulties; and (3) explore the challenges faced by humanitarian authorities/actors in implementing these measures.

## Methods

A descriptive qualitative study was conducted to understand a complex and detailed phenomenon from the meanings that people who experience it give to it [20].

### Research setting

The study was conducted in Burkina Faso, in the commune of Kaya, the capital of the Centre-North region. As of July 31, 2021, the number of IDPs in the region was estimated at 483 546 [6].This region had the second-largest number of IDPs among the 13 regions of the country. In addition, the commune of Kaya had the largest number of IDPs during the same period, with 123,610 people [18]. The majority of IDPs are children (58.71%), women (25.6%), and men (16.4%). Their care is coordinated by the decentralized services of the Ministry of Humanitarian Action with the support of several NGOs that directly or indirectly support the state. In Kaya, IDPs live either in host households or in accommodation sites.

### Identification of participating sites

The Regional Directorate of Gender, National Solidarity, Family, and Humanitarian Action of the Centre-North was the entry point for this study. Its main role was to help us understand the organization of the IDP sites chosen for this study. Following an interview, the research team was directed to the provincial directorate of social action, which facilitated contact with their delocalized agents, generally in pairs for each site they manage. In the city of Kaya, the IDPs are installed by affinity according to the localities of origin and by wave of arrival. They are grouped in 13 sites located mostly on the outskirts of the town, particularly in undeveloped areas or informal settlements. These provincial directorate agents are supported by a committee of ten people representing the IDPs and the host populations. All activities carried out on the sites are organized with the support of this local committee.

Study participants were recruited from two sites, based on the housing conditions of the IDPs: tents provided by the High Commission for Refugees (UNHCR) or constructed houses. Considering ongoing research or intervention activities in the sites, the research assistants were connected with the management committee of each site to recruit respondents. The social workers ensured that other activities from different structures were not going on at the same site at the same time to ensure IDPs or the people working on securing the sites were not overloaded.

### Description of study sites

The two IDP sites selected had different profiles. In Site 1 (located in the Nabakoaga neighborhood), the majority of IDPs were housed in rented houses or houses they had built themselves on land granted by the indigenous population. IDPs were scattered and often confused with the indigenous population. Some IDPs even bought land to build on. This site had been housing IDPs for over 3 years. It is managed by two agents of the social action services.

In Site 2 (located in Sector 6), IDPs were housed mostly in tents built by UNHCR. This site was smaller in size and had been housing IDPs for one year. Compared to Site 1, the IDPs at Site 2 were more crowded and overcrowded. The site parameter was fenced, however, the fence tended to disappear because of the high number of IDPs, leading some to settle in areas outside the designated fenced site. Site 1 was mainly inhabited by Mossi and Foulsés, while on site 2, in addition to Mossi and Foulsés, there were also Fulani. Several humanitarian NGOs were working on both sites.

### Selection of participants

Two categories of respondents participated in this study: IDPs and authorities and/or humanitarian actors involved in the care of IDPs. At each site, the selection of IDPs was based on criteria such as the order of arrival of IDPs at the interview site, age, gender, marital status, and origin of IDPs. When two IDPs had almost the same socio-demographic characteristics, only one IDP (the first) was selected for the interview to diversify the sample.

The stakeholders were chosen according to their availability and involvement. Humanitarian actors were selected among agents and managers of NGOs and local associations working in the humanitarian field (*n* = 8), while social and health authorities were selected from among agents of the provincial directorate of social action, health agents who worked in the care of IDPs in the sites, community health agents, and staff of the Kaya health district (*n* = 10). A heterogeneous sample of 29 IDPs and 18 humanitarian actors, including 26 men and 21 women, was obtained. Of the IDPs and humanitarian actors, 20 had no education. The average age of the IDPs was estimated at 38.4 years, with the youngest IDP being 20 years old and the oldest being 63 years old.

### Data collection

We conducted a total of 47 semi-structured individual interviews. The interviews were recorded with the consent of the interviewee. We triangulated the sources of data collection in order to obtain a diversity of viewpoints and a more or less complete picture of the subject under study [21]. Two flexible interview guides were used to collect the data. The first interview guide was for humanitarian actors and social and health authorities and the second was for IDPs. The themes of the interviews were: (1) the knowledge of the different actors (humanitarian actors and IDPs) about COVID-19 quarantine, isolation and lockdown measures; (2) difficulties encountered by IDPs in practicing/following the quarantine, isolation and lockdown measures; (3) challenges faced by humanitarian actors in implementing quarantine isolation and lockdown measures; and (4) adjustments made by IDPs and humanitarian actors in implementing quarantine isolation and lockdown measures. Data were collected between June 21 and July 30, 2021, by four research assistants trained in sociology.

### Data analysis

The transcripts were imported into the NVivo11© software for a content analysis [22] guided by a mixed coding method combining the inductive and deductive approach [23]. This choice is justified by our desire to leave room for emerging themes since the codification will be more or less free even if we had the elements of our analytical framework in mind. Based on the objectives of the study and the themes developed in the interview guides, a non-rigid coding guide was drawn up. An open coding of 4 interviews was carried out by a research assistant, then discussed by the qualitative research team to clarify certain codes and check their plausibility with the coded segments (verbatims). The rest of the interviews were then coded, making room for emerging codes. The analysis followed a four-stage reasoning approach: (1) identifying text extracts with meaning in relation to the research objectives and questions, (2) coding these extracts by affixing a representative theme to them, (3) grouping the themes into categories that highlight the trends emerging from the results, using an iterative process of constant comparison, and (4) consolidating the development of a grouping model for the emerging categories, by means of a hypothesis formulation and conclusion testing exercise, until data saturation was reached [22]. The interpretation adopted was the exploratory analysis method (general inductive analysis) to examine and synthesis the data, i.e., the units of meaning coded and grouped into categories according to the research objectives.

### Ethics and confidentiality

The study was approved by the ethics committee of Laval University under approval number 2020 − 256/24-11-2020 and by the health research ethics committee of Burkina Faso under number 2020-0-152. Informed consent was obtained from each participant after explaining the objectives of the study and the risks involved. Particular care was taken to respect the norms of confidentiality and non-disclosure of the participants’ identities. In reporting participants’ responses, identifying information was removed and participants assigned pseudonyms based on the type of participant, gender, and project site. Project personnel signed a confidentiality and ethical practices agreement. During the data collection, the research assistants respected the barrier measures (hand washing, nose covering, physical distancing).

## Results

### Isolation and quarantine measures

#### Knowledge of isolation and quarantine measures

Most IDP respondents had little knowledge of isolation and quarantine measures. The majority of respondents believed that if a person was suspected or ill, they should contact the health authorities. Only health workers could determine the status of suspected persons and ensure their management. They also explained that they had been informed of the existence of a toll-free number to call when a close person showed symptoms of the disease. For some, the care of sick people was done in the health centers in Kaya and for others in Ouagadougou. Health workers adopted barrier measures to protect themselves and sick people were isolated. One IDP explained that:

***“…****We put the people in a house in Ouagadougou, we give them food and we look after them. The nurses put on muffs and gloves, at least that’s what we saw on TV. This is what the nurses have adopted so that if you contract the disease, they can get close to you to treat you*.” (Female IDP site1).

They were aware, however, that isolation or quarantine involved the removal and seclusion of individuals to prevent the spread of the disease. Although positive or suspected cases were not officially reported or recorded among IDPs during the period of our data collection, IDPs were aware of the basic principles of isolation and quarantine, which is to avoid contact with a suspected or COVID-19 positive person.

Some IDPs, however, had “good” knowledge of isolation and quarantine measures. For them, isolation and quarantine measures are applied to suspected or COVID-19 positive persons to prevent them from infecting others. They explained that in case of doubt, it was best to avoid physical contact with suspected case, which implied not spending the night with him or her and not eating his or her leftover food. Also, the suspected person should stay at home and not expose others until the doubt is removed, i.e., until they are sure they are not a carrier of the virus. As one of them stated:

*“Being suspicious if you don’t quarantine him, by the time it’s proven to be true, he’s already infected you. So, you must quarantine him at the time of suspicion. Somebody should not be sick, and you come in, you touch him all over, you eat the rest of his food, and you sleep with him, no. From the moment he is not well, even if it is a cold, you must get away from him. Even if it’s a matter of suspicion you must quarantine him because if you don’t quarantine him and then you realize it’s true, by now he would have already infected enough people and it’s going to be harder to treat now because it would be several people who will have the disease.”* (Male IDP site 1).

The majority of IDPs and humanitarian actors felt that quarantine was an effective way to fight the pandemic. Consequently, if suspected cases turned out to be positive, then many people would have been prevented from possible exposure and infection. The same was true for isolation. They explained that in Africa, and specifically in Burkina Faso, visits were seen as a sign of consideration and solidarity towards the sick. It is also the manifestation of moral and psychological support to the suffering person. As COVID-19 is a contagious disease, visits to the sick became very dangerous for visitors who could easily get infected. Thus, isolation and quarantine measures prevented visits from close relatives and friends. This helped prevent transmission and slowed down the spread of COVID-19 within the community. One respondent explained that:

*“At this level, we know that it helps to limit the disease if we put the patient in isolation. At home in Burkina Faso, if a person is sick, we will go and see how they are doing! If you don’t visit them when it’s a breathing disease, you can’t be contaminated! So, we know that isolation is a good measure, otherwise, people will get infected.”* (Male IDP site 1).

In summary, isolation and quarantine were perceived by respondents as effective measures to control the spread of COVID-19. These measures, according to them, limited the number of cases of contagion through the observation of people who were ill or suspected of being carriers of the disease. According to the respondents, without the isolation and quarantine measures, the disease would have spread widely in the city of Kaya and among IDPs because of the solidarity and moral support visits.

#### Isolating or quarantining oneself in case of need: difficulties perceived by IDPs

IDPs were concerned about the application of isolation and quarantine measures in their homes. The first difficulty they identified concerned the operationalization of these measures. The question and difficulty of the physical space that would serve as a quarantine and/or isolation site was highlighted. Indeed, the lack of housing was already a problem in the case of IDPs before the advent of the pandemic and was becoming more acute with the continuous arrival of newly displaced persons to Kaya. There were no rooms available in accommodation site to isolate or quarantine positive or suspected cases and it was also difficult to find premises to rent that would serve as isolation or quarantine sites because of the shortage of housing in the city. Additionally, respondents felt that landlords would be reluctant to have their homes used as quarantine or isolation sites because of the risk of not finding tenants. One IDP explained that:

*“It is the acquisition of the house! To have a house is already difficult. And you are looking for a house for a sick person! If the patient has no one to help him find a house, he will look for a house for a long time before he finds it. Because here we don’t find houses easily.*” (Female IDP site 1).

In addition to the housing deficit, most respondents mentioned that the need for food support increased for isolated or quarantined individuals and members of households for which they were responsible. They explained that the living conditions of IDPs required them to travel either to work or to humanitarian institutions to seek “daily sustenance”. Indeed, IDPs were not fully taken care of by social services and humanitarian actors. Social transfers (food, money, clothing) only covered part of the IDPs’ necessities. Thus, non-beneficiaries and partial beneficiaries were unable to quarantine themselves or isolate themselves if suspected or positive for COVID-19, especially when they were the pillar or main provider of the household. One explained that:

*“… we who came here, there are people who get food and there are people who don’t. If you don’t have it, you have to go out and get it… if you are an active person and you have to bring food for others, you are the pillar. It’s complicated for you and your family if you have to be forbidden to go out.*” (Female IDP site 1).

Although some IDPs recognized the need for, and effectiveness of, these measures, they admitted that placing a family member in isolation or quarantine was a real dilemma given their social and economic context. Some explained that not visiting and/or approaching a sick relative could be a source of social disruption. For them, it could appear as an abandonment, a refusal of assistance that could lead to discord and the breaking of social ties.

*“Isolation is good on the one hand, but on the other hand is not good. Because if you isolate a person, they will say that their loved ones have abandoned them because of their illness. They will say that they have run away and left him alone with his illness and that they don’t want to help him. But this is not true. As it is said everywhere that the disease is bad, the relatives did not run away. We want the disease to heal at home before we come, to be together, otherwise, we have not run away. The difficulties that are in isolation are a problem. Your brother from the same mother, we come to say that since he has covid-19, you have to isolate him. We can isolate him, but we won’t be happy…*” (Female IDP site 1).

The IDPs who felt there were no difficulties if isolation or quarantine were required supported their views with two main arguments. First, these individuals had previous experience in their communities with diseases such as measles that require isolation and removal of a sick person to protect other community members. For them, experience with these diseases could facilitate the acceptability, understanding, and implementation of these measures. The other argument was that if the other members of the household and the person concerned were aware of the danger of contagion and the risk involved, they could find a place to isolate and/or quarantine the person. The sick person would not feel excluded, but rather satisfied that they did not have to put other members of their household and the host site at risk. For them, it was more of a challenge to make the IDPs aware of the risk involved. One explained that:

*“…looking for a place to place him and taking care of his health so that family members don’t come near him is not hard enough. Because he knows that if he goes near people, it will not be him alone, but many people. If the sick person knows that he could infect others by staying in physical contact with those around him, he can understand and accept the isolation. His family members will agree and be happy.*” (Male IDP site 1).

##### Coi

ncidently, some humanitarian actors, particularly health authorities, felt that isolation and/or quarantine would not be a problem if needed. They explained that the health authorities were aware of the challenges of dealing with IDPs if quarantine and/or isolation were necessary. Thus, in the health district’s response plan, facilities had been identified for this purpose. One health worker explained that:

*“… the district had already identified premises for this. If there are cases of displaced persons, as these are people who are in an advanced state of precariousness, it will be a bit complicated, since they may have a house with a living room where there are up to 10 or 15 people inside. So, the district had already identified premises for this, but fortunately, we haven’t had any cases here… I don’t think they would encounter any difficulties, since everything was planned at the district level in their response plan.” (*Male, Health worker).

#### Adjustments to perceived difficulties

Health authorities claimed to have a strategy for managing positive and suspected cases. However, there were no positive or suspected cases reported at IDP sites during the period of this study. Therefore, IDPs did not face these different measures of isolation or quarantine. For them, suspected and positive cases should be referred to health centers for management. Most health workers had very little idea of the possibility of having logistical means for quarantining suspected or positive persons at the sites. Others, however, argued that specific tents could be set up. This possibility was only considered for suspected individuals. One argued that:

*“…well, that’s what I was saying earlier that it’s good to always want to find places, houses for that. If it is difficult, we can erect tents. The person who is suspected or confirmed stays there. If during the control examinations, they prove to be negative, they go back home.*” (Male, Health Agent).

For others IDPs, the adjustment will consist of respecting the required instructions to stay away. This means that people close to or living at the site must take steps to avoid a person who is suspected or tested positive for COVID-19. But respondents said that to reduce frustration, this required that, at the end of the isolation or quarantine, they apologize to the persons concerned while explaining the rationale for their attitude. This would open a discussion to prevent or resolve possible frustrations caused by the isolation and lack of visits from friends and relatives, but also to make people understand the objective of protecting the population and curbing the spread of the disease. The apology was seen as reparation for what could be interpreted by the patient or suspected case as abandonment. In this way, social ties could continue.

“… *it would be necessary that when the person is cured, that I go to ask for forgiveness and to let her know that it is because they said that the disease is contagious that they isolated her, otherwise it is not that they abandoned her. Since the disease is over, I came back so that we could still be together. But the difficulties that can exist in the case of isolation can be the cause of discord between us because it is complicated to avoid a person. After all, he is sick. If it is going to be the occasion of discord between you, it is not good.*” (Female IDP site 1).

### On the lockdown measure

#### Knowledge of the actors on the lockdown measure

For many respondents, the implementation of lockdown measures was a strategy that health authorities developed following their awareness of the health risks related to the transmission routes of the virus. Thus, to contain the disease and avoid outbreaks, the authorities adopted lockdown measures that limited the movement of individuals between and within localities where positive cases had been identified. They viewed curfews, inter-city travel bans, and border closures as lockdown measures that reduced physical contact between individuals and the emergence of new transmission sites. One IDP explained that:

*“What I saw was the closing of the entrances to the city of Ouagadougou, that is to say, no cars going out or coming in, no public transportation, and the establishment of a curfew. That’s what I remember. They had instituted all these measures maybe they saw that these are the elements that could cause the spread of the disease if they were not respected*.” (Male IDP site 2).

Most respondents felt that given the health situation in other countries and Burkina Faso, the lockdown was an effective response measure. They knew these measures and were aware of the need to apply them. For IDPs and humanitarian actors, the mobility of people was the primary factor that contributed to the spread of the disease. They felt that lockdown measures were effective in limiting the mobility of people. Closing land and air borders and prohibiting intercity travel limited mobility of people and helped reduce risk of exposure and spread of disease. This was the opinion of one respondent who mentioned that:

*“The fact that vehicle traffic and travel between major cities have been banned has prevented the disease from spreading. Because they can go somewhere and get the disease and make the disease travel. You can get the disease, put yourself in a vehicle and nobody knows. You can infect all the rest of the people who are in the vehicle and they’re going to bring the disease back to their town or village.”* (Female IDP site 1).

#### IDPs’ difficulties in implementing lockdown measures

The city of Kaya experienced positive cases of COVID-19 and when the first cases were reported, the city was placed under lockdown. It was therefore forbidden to leave and/or enter the city.

It is important to note that generally, most IDP men moved to other localities to pursue economic activities (i.e., labor/employment) to make money and take care of their households. Therefore, many of them tended to cross the borders of Burkina Faso, and if they did not leave the country, they went to other regions within Burkina Faso in search of work and opportunity to make money. This was one of the reasons why there were more women and elderly people in the shelter sites. Thus, female IDPs were very mobile in the host communities while male IDPs were mobile not only in the host communities, but also throughout the different communities and regions of the country, or outside the country. With the COVID-19 confinement and closure of land borders, the ability to travel to other regions in search of labor was limited. They explained that these measures had a significant impact on their daily lives, including the provision of food for their households and their ability to send their children to school. One IDP explained:

“*Ah, we’ve encountered several difficulties! You’re not going to spend like you used to. The money doesn’t come in anymore, it doesn’t come in anymore. Those who used to go to Côte d’Ivoire don’t go there anymore… these are difficulties. There are still many difficulties. Even if you are a woman, there are still many difficulties. My husband, who is sitting here, wanted to go to Côte d’Ivoire, but if the road is closed, he cannot go anymore. Food is a problem, and being able to raise your children is a problem. Even us who came here, tell you that there is a school here at 25,000 FCFA, you sit there, and you don’t have the money, it’s a problem. You want to put your child in school, but you don’t have any* [money], *how are you going to do it? That’s a problem*” (Female IDP, site 1).

Another challenge for IDPs was the reduced ability of families to reunite following the closure of borders. Indeed, in the first villages affected by insecurity, men were the preferred targets of unidentified armed groups. This led to the migration of many men to the provinces or countries bordering Burkina Faso, leaving their families either in the villages or in the host site in hopes of reuniting with them later. This was also the intention of IDP men who, due to the precariousness of life in the host sites, migrated to neighboring localities or countries to engage in income-generating activities or to seek work.

The confinement made it difficult to attempt family reunification, but also to trace families who lost touch during periods of displacement. One respondent explained:

*“The IDPs who arrived here, the able-bodied, went to grow vegetables where there are dams. Others trade and move from village to village. Well, they used to do different activities. Now, because of the confinement, where you find yourself, you stay there. For example, an IDP man who is there and ends up in Boromo for his commercial activity, the confinement has made it impossible for him to come. If there are difficulties in his family in Kaya, who is going to manage? Do you see? Some people have fled, and their wives don’t know if they are living or not, they are here with the children. Some people recognize that in any case their husbands are no longer there, they take their responsibilities. But the women whose husbands have fled, we don’t even know where they are, it’s a bit tricky with the confinement to hope to get news*” (Male, Health worker).

However, we note that no difficulties related to the curfew were highlighted by IDPs and health workers. It is possible that respecting the curfew had no impact on their daily lives.

#### IDP adjustments to lockdown measures

The main adjustment of the IDPs was their commitment to compliance and awareness of the lockdown measures. This commitment was based on the perceived opportunity for them to comply with these measures. It was an opportunity for them in that they were not exposing themselves, their household members, or other IDPs to the risk of contracting the disease. Some explained that they were involved in sensitization to get IDPs not to expose themselves, their household members, and others. Thus, the message was to take steps not to be the person through whom the disease would pass to affect others. The IDP leaders had even demanded from their peers that they limit their mobility not only to protect themselves but for the benefit of the entire community. One of them explained:

“*That’s why we asked everyone to stay at home and when the spread of the disease decreased, we allowed everyone to go about their business. It is so that you don’t walk around so that you don’t enter a town and catch the disease and then spread it to everyone and they will say that it is such and such who brought the disease. For example, when you leave here for Kalambaogo, from there you go to another town. If there is no disease in this locality, you say to yourself that you are not sick, you will come and spread it…* (Female IDP site 1).

### Challenges faced by the authorities in implementing lockdown, quarantine, and isolation measures

Communication was one of the biggest challenges facing humanitarian actors. The goal of communication was to make IDPs aware of the risks of the pandemic. To meet the communication challenges and get IDPs to comply with the response measures, the authorities thought it was necessary to solicit the involvement and support of IDP community leaders. They explained that if the measures were imposed, IDPs would do so as a courtesy to their presence but would not respect the measures once the humanitarian actors had left. Hence the need to engage leaders among the IDP communities. These leaders act as relays in the sites and discuss with the IDPs the importance of respecting these measures not only for their health, but also for the health of all the people who are on the site. Thus, to ensure a certain level of adherence by IDPs, humanitarian actors involved IDP leaders in their communication strategies through the committees set up in each IDP site. One humanitarian actor stated:

*“ … We need to work in advance with community leaders, religious leaders, all these people. There needs to be communication so that it can be passed on. If it doesn’t go through them at their level, it will be very complicated. Because I know that, for example, to get up and say that there is a disease, covid-19, respect the barriers without having worked with these resource persons beforehand, it will be very complicated. And it’s enough that one person refuses and the whole family refuses.”* (Health worker).

Humanitarian actors also highlighted the difficulties associated with caring for IDPs if they were to be placed in isolation or quarantine. In the case of IDP isolation, they explained that while medical care was provided, food and access to shelter (a house) could be a problem. This echoed the concern raised by IDPs about their care in the event of isolation or quarantine. Other humanitarian actors also highlighted the psychological state of IDPs. They explained that IDPs were already weakened by their experiences due to the security crisis. They had experienced traumatic situations and had feelings of fear. Under these conditions, isolating them to comply with COVID-19 measures could exacerbate this trauma. The challenge for them was to find a way to put IDPs in isolation or quarantine without exacerbating their trauma.

“*It’s double trauma only. It’s simply because these are people who are already traumatized. That’s one. These are scared people. These are people who were in their village and when you want to bring them to other conditions, it’s difficult. They won’t understand and that’s what makes it difficult.*” (Education advisor and association president).

## Discussion

This study is one of the first to qualitatively analyze not only the difficulties IDPs face in implementing lockdown, isolation, and quarantine measures, but also the challenges faced by humanitarian actors and administrative authorities in Kaya, Burkina Faso.

Findings revealed a lack of clarity and coordination for humanitarian actors, public administrators and IDPs in terms of responsibility in managing suspected or confirmed cases during isolation or quarantine. Some humanitarian actors claimed that arrangements had been made for effective and efficient care when needed, yet the fear of not having sufficient necessities (housing, food support) for the isolation of the sick or quarantine of suspected cases remained a concern for them. This concern was also shared by IDPs but contrasted with public administration actors who mentioned that facilities had been identified and prepared to isolate and quarantine IDPs if necessary. This reveals a communication problem, as IDPs thought that isolation and quarantine were their responsibility, even though they lived in shared accommodations/spaces, did not have facilities at the sites or the income to implement these measures. The loss of their homes and possessions due to forced displacement positioned them in a precarious housing condition [24].

The lack of communication and understanding of responsibility, compounded by lack of resources to isolate/quarantine, lead to under reporting of cases and increased risk of disease spread.

Recent work in Mali and Congo also highlighted the lack of facilities for quarantine and isolation, physical space to build new facilities, and financial resources to support IDPs during isolation and quarantine [10, 12, 24, 25].

In addition, humanitarian and administrative actors mentioned that the application of isolation and quarantine could exacerbate the trauma and psychological distress experienced by IDPs because of the violence they had experienced. The routes IDPs took to reach safe areas were often rough and circuitous, passing through several temporary living quarters before arriving at their camps. Additionally, IDPs were dealing with cultural grief (grief over the loss of cultural identity and social systems), traumatic and/or ambiguous loss of loved ones, fractured families with changing roles, acculturative stress, and uncertainty (of safety, housing) that exacerbate negative mental health [26–28]. Thus, the seclusion caused by isolation and quarantine could be perceived by IDPs as a sign of abandonment and lack of compassion Consequently, Claude et al. [24] describe how social isolation to prevent COVID-19 elicited feelings of hopelessness and abandonment by IDPs in Congo.

The contrast of isolation/quarantine measures with IDPs’ values of solidarity and reciprocity in social interactions was highlighted. The IDPs came from rural areas and are a people united by strong social ties where assistance, compassion, along with moral and psychological support to one of their own in case of illness is a moral duty.

The isolation due to illness was a source of psychological suffering according to the respondents since isolation or quarantine created a feeling of exclusion and abandonment that exacerbated the trauma experienced by these people when they were forced to leave their villages due to the terrorist violence [29–31].

Finding revealed that the lockdown measures reduced IDPs’ mobility and contributed to their financial vulnerability because household heads who migrated to other locations in search of income-generating activities were no longer able to do so. Additionally, some IDPs who were the main providers of household income were trapped away from the households. For the respondents, this precarious situation was perceived to be harsher than contracting COVID-19. Similarly, in India workers stranded on their way home or stuck in slums without income or food due to unannounced confinement during COVID-19 told the media that they would starve to death before the virus reached them [32, 33].

During the time of this research project, isolation and quarantine did not take place at IDP sites studied, yet respondents identified difficulties and challenges associated with these public health measures, highlighting key IDPs vulnerabilities. To mitigate the higher risk of social and health inequities faced by IDPs, public administrations (i.e., ministries of health) and humanitarian actors should be prepared and implement planned risk communication, community engagement and social mobilization (RCCE) activities [34, 35]. It is important to raise awareness, educate and engage in health promotion regarding pandemic and epidemic risks and preparedness within IDP communities, while also engaging them as responsible stakeholders within their local context to combat misinformation [36]. RCCE activities should be a collaboration effort between humanitarian actors, ministries of health and the community to ensure localization (i.e., local context is considered) as well as consistent and clear messaging about implementation and responsibility of health and safety measures [35].

The Burkinabe authorities should also recognize that the living conditions of IDPs make them very vulnerable to the spread of infectious diseases. Following the African Union Convention for the Protection and Assistance of Internally Displaced Persons in Africa [37] - to which Burkina Faso is a party – state parties, international organizations and humanitarian agencies have an obligation to ensure protection and assistance to IDPs. According to UNHCR, 80% of the world’s refugee population and almost all IDPs live in low- and middle-income countries, many of which have fragile health, water, and sanitation systems [38]. Nearly 2 million IDPs in Burkina Faso live in informal settlements where they are vulnerable to disasters, have very few resources, and have little resilience [6]. IDPs experience socio-economic vulnerability, due their precarious housing, associated negative mental and physical health outcomes, and food insecurity related to their displacement [39–43]. This situation of vulnerability could therefore make them more vulnerable to the collateral effects of COVID-19. Under the convention, the state must consider and is obliged to address these needs with special protection, as would happen in the case of a lockdown.

This study has strengths and weaknesses. In terms of strengths, to the knowledge of the authors, this is the first research in Burkina Faso that addresses isolation, quarantine, and confinement among IDPs. The use of the individual interview method provided an in-depth account of respondents’ lived experiences and views. However, this research does not claim to be representative of Burkina Faso’s IDPs. Although information saturation was reached, we recognize that future research raising similar issues would validate, build on or compare our findings. Additionally, the absence of reported cases of COVID-19 in the IDP population studied is a limitation since respondents could not describe lived experiences of isolation and quarantine. Rather, they shared their perceptions and apprehensions of what might arise as difficulties and challenges related to isolation. Nonetheless, this anticipatory thinking combined with the lived experience of lockdown has produced knowledge that will inform decision-making in the event of future outbreaks, conflicts, emergencies, or health crises that may lead to isolation and lockdown.

## Conclusion

The purpose of this study was to document the knowledge, challenges, difficulties, and adjustments encountered by humanitarian actors and IDPs in implementing lockdown, isolation, and quarantine measures in the context of COVID-19 in the North Central Region of Burkina Faso. Findings suggest that precariousness of living situations of IDPs, their vulnerability to existing negative social and health outcomes, as well as cultural norms must be considered for effective implementation of isolation, quarantine, and lockdown measures at IDP sites. Additionally, partnership and collaboration amongst state and local actors regarding emergency preparedness, as well as appropriate risk communication and community engagement are needed to mitigate challenges to implementation and ensure relevance to IDPs who should be key stakeholders in this work. The knowledge gained from this study informs the management of future health crises among IDPs.

## Data Availability

Full transcripts cannot be shared publicly due to potentially identifying information. The content and words of respondents in interviews could potentially be used to identify individuals. Even anonymisation could pose risks to confidentiality. Data are available upon request to the Comité d’éthique pour la recherche en santé in Burkina Faso (+ 226 72757187).
